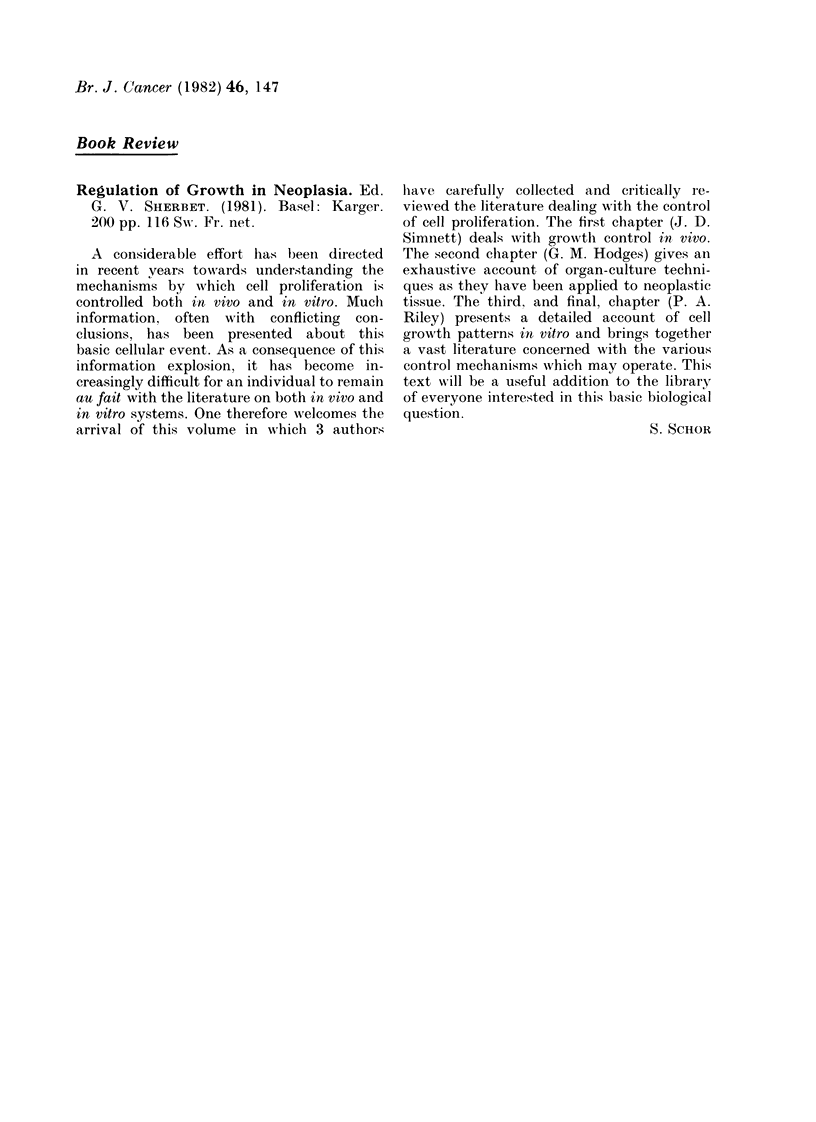# Regulation of Growth in Neoplasia

**Published:** 1982-07

**Authors:** S. Schor


*Br. J. Cancer* (1982) **46**, 147

### *Book Review*

**Regulation of Growth in Neoplasia.** Ed. G. V. SHERBET. (1981). Basel: Karger. 200 pp. 116 Sw. Fr. net.

A considerable effort has been directed in recent years towards understanding the mechanisms by which cell proliferation is controlled both *in vivo* and *in vitro*. Much information, often with conflicting conclusions, has been presented about this basic cellular event. As a consequence of this information explosion, it has become increasingly difficult for an individual to remain *au fait* with the literature on both *in vivo* and *in vitro* systems. One therefore welcomes the arrival of this volume in which 3 authors have carefully collected and critically reviewed the literature dealing with the control of cell proliferation. The first chapter (J. D. Simnett) deals with growth control *in vivo*. The second chapter (G. M. Hodges) gives an exhaustive account of organ-culture techniques as they have been applied to neoplastic tissue. The third, and final, chapter (P. A. Riley) presents a detailed account of cell growth patterns *in vitro* and brings together a vast literature concerned with the various control mechanisms which may operate. This text will be a useful addition to the library of everyone interested in this basic biological question.

S. SCHOR